# Use of Nonsteroidal Anti-Inflammatory Drugs and Risk of Breast Cancer: Evidence from a General Female Population and a Mammographic Screening Cohort in Sweden

**DOI:** 10.3390/cancers15030692

**Published:** 2023-01-23

**Authors:** Kejia Hu, Maria Feychting, Donghao Lu, Arvid Sjölander, Kamila Czene, Per Hall, Fang Fang

**Affiliations:** 1 Institute of Environmental Medicine, Karolinska Institutet, 17177 Stockholm, Sweden; 2 Department of Medical Epidemiology and Biostatistics, Karolinska Institutet, 17177 Stockholm, Sweden

**Keywords:** breast cancer, nonsteroidal anti-inflammatory drug, aspirin, mammographic density

## Abstract

**Simple Summary:**

Non-steroidal anti-inflammatory drugs (NSAIDs) are a group of commonly used drugs which target inflammation. Because inflammation is a critical component in cancer development, NSAID was proposed to reduce the risk of breast cancer by some studies. However, the results are inconsistent between studies. Moreover, there are insufficient data regarding risk of breast cancer with different characteristics, for example cancer subtype and stage, and few studies have investigated whether the risk will differ by breast density or previous breast disorders. Therefore, we investigated the association between use of NSAIDs and risk of breast cancer using data on NSAID use and breast cancer diagnosis from women in Sweden in general and women from a breast cancer screening program. Overall, we did not have strong evidence to support an association between the use of NSAIDs and the risk of breast cancer. More studies in diverse demographic and geographical settings are needed to confirm our findings.

**Abstract:**

A link has been proposed between the use of nonsteroidal anti-inflammatory drugs (NSAIDs) and the risk of breast cancer. There is, however, insufficient data regarding the subtype and stage of breast cancer, and few studies have assessed the interaction between the use of NSAIDs and breast density or previous breast disorders. There is also a lack of data from population-based studies. We first conducted a nested case-control study within the general female population of Sweden, including 56,480 women with newly diagnosed breast cancer during 2006–2015 and five breast cancer-free women per case as controls, to assess the association of NSAID use with the risk of incident breast cancer, focusing on subtype and stage of breast cancer as well as the interaction between NSAID use and previous breast disorders. We then used the Karolinska Mammography Project for Risk Prediction of Breast Cancer (Karma) cohort to assess the interaction between NSAID use and breast density in relation to the risk of breast cancer. Conditional logistic regression was used to estimate the hazard ratio (HR) and a 95% confidence interval (CI) was used for breast cancer in relation to the use of aspirin and non-aspirin NSAIDs. In the nested case-control study of the general population, exclusive use of aspirin was not associated with the risk of breast cancer, whereas exclusive use of non-aspirin NSAIDs was associated with a modestly higher risk of stage 0–2 breast cancer (HR: 1.05; 95% CI: 1.02–1.08) but a lower risk of stage 3–4 breast cancer (HR 0.80; 95% CI: 0.73–0.88). There was also a statistically significant interaction between the exclusive use of NSAIDs and previous breast disorders (*p* for interaction: <0.001). In the analysis of Karma participants, the exclusive use of non-aspirin NSAIDs was associated with a lower risk of breast cancer among women with a breast dense area of >40 cm^2^ (HR: 0.72; 95% CI: 0.59–0.89). However, the possibility of finding this by chance cannot be ruled out. Overall, we did not find strong evidence to support an association between the use of NSAIDs and the risk of breast cancer.

## 1. Introduction

Anti-inflammatory drugs may influence the risk of cancer development due to the important involvement of inflammation in carcinogenesis [1,2]. Nonsteroidal anti-inflammatory drugs (NSAIDs), due to their role in inhibiting the activity of cyclooxygenase (COX), have therefore been examined in a substantial number of studies as a potential risk or protective factor for different cancer forms, including breast cancer [3,4,5,6,7,8,9,10,11].

Existing meta-analyses [6,7,8,9,10,11] suggested that the use of aspirin and non-aspirin NSAIDs was associated with a lower long-term risk of breast cancer, although most of the evidence concerns aspirin (Table S1). Such association was, however, not supported by a randomized clinical trial within the Nurses’ Health Study [3,4], large-scale cohort studies [5,12], and recent meta-analyses of only cohort studies [9,10]. Some studies reported that the association was restricted to specific groups of women, such as overweight [13] or postmenopausal [14] women, or women with other pharmaceutical interventions (e.g., hormonal therapy [15] or proton-pump inhibitors [14]). It remains inconclusive if and how the association would differ by molecular subtype or stage. For example, several studies reported a greater risk reduction for hormonal-positive tumors [9,16,17] than for other subtypes, while others suggested a greater risk reduction [18] or a risk increment in hormonal-negative tumors [19], concerning NSAID use. Regarding stage, one study [16] reported a reduced risk of breast cancer with the use of NSAIDs except for stage 3–4 breast cancer, while another suggested an increased risk of breast cancer, especially nonlocalized tumors, among ibuprofen users [19]. Breast density measures the nonfat, radiologically dense tissue in the breast, and extensive density is an established risk factor for breast cancer [20]. Therefore the role of breast density linking NSAIDs and breast cancer risk has been examined. For instance, a large screening cohort suggested that aspirin use was associated with lower breast density [21], although another cohort study [22] failed to confirm such an association and did not find an interaction between aspirin use and breast density in relation to breast cancer risk. As benign breast disorders may also influence the risk of breast cancer [23], two other studies suggested a protective role of aspirin and non-aspirin NSAIDs in relation to the risk of breast cancer among women with benign breast disorders [24,25].

To address these inconsistencies in the literature, we took advantage of the Swedish National Register data, together with a rich questionnaire and mammographic data from the Karolinska Mammography Project for Risk Prediction of Breast Cancer (Karma) cohort, to examine (1) whether NSAID use, either aspirin or non-aspirin, reduces the risk of breast cancer, (2) whether the association differs by stage and molecular subtype of breast cancer, and (3) whether breast density and a history of breast disorders modifies the association, using a nested case-control design.

## 2. Materials and Methods

### 2.1. Study Population

In the first analysis, we used data from multiple national registers in Sweden, including the Total Population Register [26], the Cancer Register [27] (including all malignancies diagnosed in Sweden since the year 1958), the National Quality Register for Breast Cancer (Swedish acronym NKBC) [28,29] (including newly diagnosed breast cancer in Sweden since 2008, with 98% coverage compared to the Cancer Register), the Patient Register [30] (covers all inpatient care since 1987 and all outpatient care since 2001), and the Prescribed Drug Register [31] (including all prescribed drug use since 1 July 2005). The study population consisted of all women who were born before 1 July 1987, and free of breast cancer on 1 January 2006 (N = 3,485,075). We used a new-user design [32] to avoid prevalent user bias by excluding 653,072 women who used NSAIDs during an antecedent washout period of six months [33] (i.e., women had to be free of NSAID use between 1 July 2005 and 31 December 2005). The remaining 2,832,003 women were followed from 1 January 2006, until a first diagnosis of breast cancer (through the Cancer Register), death, emigration, or 31 December 2015, whichever came first, using the unique Swedish personal identification number as a key to link data between different registers.

To study the interaction between NSAID use and breast density in relation to the risk of breast cancer, we performed a second analysis using the Karma cohort, which includes 70,872 women who attended a national mammographic screening program (between ages 40 and 74) or clinical mammography between January 2011 and March 2013 at any of the four mammography units in Sweden [34]. In this study, we included only women who were free of breast cancer and were of mammographic screening age in Sweden (40–74) at their time of enrollment into Karma (N = 65,898). We excluded women who were diagnosed with breast cancer within 90 days after enrollment (N = 263) to reduce the risk of including prevalent cases. Similarly, with the analysis in the general population, we excluded prevalent users with NSAID use during a six-month antecedent washout period (N = 9970) before enrollment. Through linkage to multiple national registers, these women were followed from enrollment into Karma until a diagnosis of breast cancer, death, emigration, or 31 December 2019, whichever came first.

### 2.2. Nested Case-Control Design

**Figure 1:**
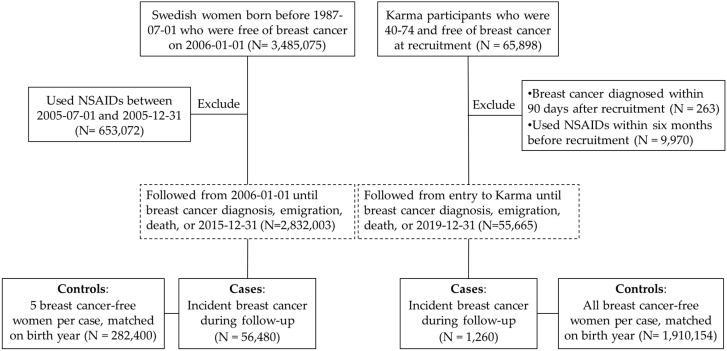
Flowchart of the study. Numbers in controls do not reflect unique individuals because individuals could be included in more than one risk set.

Due to the large sample size and time-varying nature of exposure (i.e., prescribed use of NSAIDs) and covariates (e.g., education and income), we designed a nested case-control study within the cohort of the general female population. We first identified cases of all women with an incident breast cancer diagnosed during the follow-up. For each case, we randomly selected five controls who were free from breast cancer at the diagnosis date of the case (index date), matched to the case on birth year, through incidence density sampling [35] (Figure 1). In the analysis based on Karma participants, we also used all women with an incident breast cancer diagnosis as cases. We matched each case to all women in the cohort who had the same birth year as the case and were free of breast cancer at the case’s date of diagnosis. The date of diagnosis of the case woman was used as the index date for her control women.

### 2.3. Ascertainment of NSAID Use

Through the Prescribed Drug Register, we identified the use of any NSAIDs (ATC codes: N02BA01, B01AC06, N02BA51, and M01A) between 1 July 2005 and 180 days before the index date to avoid reverse causation, as a previous study [36] suggested a lag time of six months should be used for most drug–cancer associations. We first checked if there were any filed prescriptions for aspirin (ATC codes: N02BA01, B01AC06, or N02BA51) or non-aspirin NSAIDs (ATC code: M01A) during this period, and then summarized the cumulative defined daily dose (DDD) for each category to document the relative therapy intensity with various drugs in that category. We also identified the last dispensation date of the corresponding prescription during this period and classified women according to the recency of aspirin or non-aspirin NSAID use. Previous use was defined as the last dispensation being more than one year before the index date whereas recent use was defined as the last dispensation being within 180 days to one year before the index date.

### 2.4. Covariates

In the nested case-control study of the general female population, we identified from the Cancer Register the stage of breast cancer diagnosis based on T, N, and M stages, using the 7th edition of AJCC anatomic stage groups [37]. We also identified any diagnosis of malignancies prior to the breast cancer diagnosis (since 1958), excluding nonmelanoma skin cancer. From NKBC, we identified information on the expression of estrogen receptor (ER), progesterone receptor (PR), and human epidermal growth factor receptor 2 (HER2) through immunohistochemistry (IHC) or gene copy of HER2 by an fluorescence in situ hybridization (FISH) test, among women diagnosed since 2008 (N = 46,428) (Supplementary Materials).

Via linkage with the Longitudinal Integrated Database for Health Insurance and Labour Market Studies (Swedish acronym LISA), we extracted information on education and disposable income [38]. Breast disorders before the index date were identified through the Swedish Patient Register using Swedish revisions of the International Classification of Disease (ICD) codes (ICD10: N60–N64, ICD9/8: 610–611). The Charlson Comorbidity Index (CCI) and potential indications for NSAID use (e.g., cardiovascular disease, musculoskeletal diseases, systematic inflammatory diseases, and pain and fever) were also identified from the Patient Register from the year 2000 until the index date. We identified the number of biological children and their age at first childbirth through the Swedish Multi-Generation Register [39] (with information on familial links for individuals born since 1932). Through the Swedish Medical Birth Register [40] (with information on all deliveries since 1973), we supplemented data on age at first childbirth and further identified a few other factors before the index date among a subset of women who ever gave birth in Sweden after 1982 (N = 1,258,486, 44%), including smoking status before (since 1998, N = 641,786) or during (since 1982) early pregnancy, as well as weight (1982–1989 and since 1992, N = 1,167,210, 41%) and height (since 1982) during early pregnancy, which were used to calculate body mass index (BMI). In the case of multiple records before the index date, the highest number of cigarettes and the mean BMI were kept. Use of oral contraceptives before pregnancy (since 1982) was identified from the Medical Birth Register, and we also supplemented it with data on the prescribed use of hormonal contraceptives (i.e., oral contraceptives, transdermal patches, and implants; ATC code: G02B, G03A) through the Prescribed Drug Register (since 1 July 2005).

Given that the risk of breast cancer is likely not associated with the change in breast density but rather the baseline breast density [41], we considered only the breast density measurement at the time of enrollment to Karma in the nested case-control study of the Karma participants. We used absolute dense area, which is less influenced by BMI compared to the percent density [42]. Mammograms were analyzed using the machine learning-based STRATUS software (version 1.0) [43] and we used the mean value of the dense area (cm^2^) of the left and right mammograms, mediolateral oblique (MLO) view. We imputed the missing values of dense area (N = 1077, 1.6%) using lifestyle and reproductive factors, including age, BMI, physical activity, smoking status, alcohol consumption, number of childbirths, age at first childbirth, age at menarche, and postmenopausal status, based on data among all participants of the Karma cohort (N = 65,898). We then classified breast density into low (<9 cm^2^), medium (9–40 cm^2^), and high (>40 cm^2^) dense areas, according to the first and third quartiles of the entire Karma distribution. All information, including demographic, reproductive, psychosocial, and lifestyle factors, were collected through a comprehensive questionnaire at enrollment to Karma.

### 2.5. Statistical Analysis

We first described the characteristics of cases and controls included in the analyses of the general female population and Karma participants. We then used conditional logistic regression to assess the risk of breast cancer in relation to the use of aspirin and non-aspirin NSAIDs, both exclusively and non-exclusively. Under a nested case-control design, the odds ratio estimated by conditional logistic regression can be interpreted as the hazard ratio (HR) in the underlying cohort, from which cases and controls were drawn [44]. We also assessed the association between the dose and recency of the non-exclusive use of aspirin or non-aspirin NSAIDs and the risk of breast cancer.

Different covariates were adjusted according to data availability. In the analysis of the general female population, the match-set identifier was used as a stratum. We adjusted for demographic factors in model 1 and further adjusted for CCI, potential indications of NSAID use, number of children, and age at first childbirth in model 2. We additionally adjusted for smoking, BMI, and the use of hormonal contraceptives in model 3 among a subgroup (44%) of women with available data. In the analysis of Karma participants, we also used the match-set identifier as a stratification variable and adjusted for demographic and lifestyle factors in model 1, further adjusted for potential indications of NSAID use in model 2, and additionally adjusted for reproductive factors and CCI in model 3.

In the analysis of the general female population, we studied the heterogeneity by stages and molecular subtypes of breast cancer by fitting a separate conditional logistic model for cases diagnosed with each stage or subtype and their matched controls as a competing risk analysis using a cause-specific hazard approach. We also stratified the analysis by the presence of a previous benign breast disorder in the analysis of the general female population and by mammographic density at enrollment in the analysis of Karma participants. We further tested the statistical significance of the interactions between breast disorders/density and NSAID use in an interaction model using Wald tests.

## 3. Results

### 3.1. Baseline Characteristics

We identified 56,480 cases and 282,400 matched controls in the nested case-control study of the general female population. Cases and controls had a comparable distribution of region of birth, educational attainment, and disposable income per consumption unit (Table 1). Cases and controls were also similar in terms of potential indications for NSAID use and CCI before the index date. As expected, cases were more likely to have a previous diagnosis of breast disorder or nonmelanoma malignancies. In the analysis of Karma participants, we included 1260 cases of breast cancer and, similarly, comparable characteristics were found between cases and controls (Table S2).

### 3.2. Exclusive and Non-Exclusive Use of Aspirin and Non-Aspirin NSAIDs

In the analysis of the general female population, the use of aspirin, exclusively or not, was not associated with the risk of breast cancer in the fully adjusted model (i.e., model 2), although recent nonexclusive use of aspirin was borderline associated with a lower risk of breast cancer (HR: 0.95; 95% CI: 0.91–0.99) (Table 2). The use of non-aspirin NSAIDs, exclusively or non-exclusively, was borderline associated with a higher risk of breast cancer (HR: 1.04; 95% CI: 1.02–1.06), and the association did not differ by the recency of use. There was no association between the dose of NSAID used, either aspirin or non-aspirin, with the risk of breast cancer. These associations all disappeared after further adjustment for smoking, BMI, and the use of hormonal contraceptives (i.e., model 3). No associations were noted in the analysis of Karma participants (Table S3).

### 3.3. Analysis by Stage and Molecular Subtype of Breast Cancer

In the analysis of the general female population, the clinical characteristics of breast cancer among cases diagnosed since 2008 are shown in Table S4. In the fully adjusted model, the exclusive use of aspirin was not associated with the risk of breast cancer, regardless of the stage (Table 3). The exclusive use of non-aspirin NSAIDs was, however, borderline associated with a slightly higher risk of stage 0–2 breast cancer (HR: 1.05; 95% CI: 1.02–1.08) but a lower risk of stage 3–4 breast cancer (HR: 0.80; 95% CI: 0.73–0.88). In the analysis by molecular subtypes, we found that the exclusive use of aspirin alone was borderline associated with a lower risk of triple-negative breast cancer (HR: 0.80; 95% CI: 0.64–0.99), but not otherwise (Table S5). Exclusive use of non-aspirin NSAIDs was associated with a slightly higher risk of Luminal A (ER-positive, PR-positive, and HER2-negative) and triple-negative (ER-negative, PR-negative, and HER2-negative) breast cancer.

### 3.4. Effect Modification by Mammographic Density or Previous Breast Disorder

In the analysis of Karma participants, the exclusive use of non-aspirin NSAIDs alone was associated with a lower risk of breast cancer (HR: 0.72; 95% CI: 0.59–0.89) among women with high-density breasts (average dense area >40 cm^2^) in the fully adjusted model (i.e., model 3) (Table 4). The interaction between mammographic density and the exclusive use of NSAIDs was, however, not statistically significant (*p* = 0.06). In the analysis of the general female population, no statistically significant association was noted between the exclusive use of NSAIDs and the risk of breast cancer, regardless of previous breast disorders (Table S6). There was, however, a statistically significant interaction between the exclusive use of NSAIDs and previous breast disorders (*p* < 0.001).

## 4. Discussion

In this study, which assessed the risk of breast cancer in relation to the use of NSAIDs, we did not find evidence of an association between aspirin use and the risk of breast cancer. However, we detected a slightly higher risk of stage 0–2 breast cancer, but a lower risk of stage 3–4 breast cancer in relation to the use of non-aspirin NSAIDs. The latter was mainly observed among women with high breast density.

Our finding on a null association between aspirin use and the risk of breast cancer is coherent between the analyses of the general Swedish female population and Karma participants, after adjusting for different potential confounders. This is also consistent with results from the only existing randomized clinical trial with a median follow-up of 10 years [3] or 18 years [4], as well as other cohort studies with a large sample size [5,12]. Taken together, these results argue against the substantial benefit of aspirin in the prevention of breast cancer in general, despite its potential protective effect on other cancers [4,5,33]. We observed an inverse association between the exclusive use of aspirin and a lower risk of triple-negative breast cancer in the analysis of the general female population. Further studies are, therefore, needed to validate this observation and exclude the possibility of finding by chance.

We observed a slightly increased risk of early-stage breast cancer in relation to non-aspirin NSAID use. In contrast, non-aspirin NSAIDs were found to be associated with a lower risk of high-stage breast cancer and any breast cancer among women with high breast density. This appears at odds with a previous study reporting a slightly increased risk of breast cancer, especially nonlocalized tumors, among users of non-aspirin NSAIDs (i.e., ibuprofen) [19]. To the best of our knowledge, there is only one cohort study [22] investigating the interaction between aspirin use and breast density in relation to breast cancer risk where no interaction was found. However, this study is limited in sample size and might suffer from recall bias due to self-reported aspirin use. We extended the existing knowledgebase by demonstrating the different associations between non-aspirin NSAID use and the risk of breast cancer by cancer stage and mammographic density. Our finding on the association between non-aspirin NSAID use and increased risk of early-stage breast cancer might be partially attributable to surveillance bias, assuming that users of non-aspirin NSAIDs might be more observant regarding breast symptoms. An inverse association between non-aspirin NSAID use and a lower risk of high-stage breast cancer is, however, supported by evidence from animal models suggesting that a COX2 blockade might reduce the lymphatic metastasis of breast cancer [45]. Therefore, it is biologically plausible that non-aspirin NSAIDs may delay the progression of breast cancer to a later stage. The stronger association among women with dense breasts is further plausible due to a higher level of COX-2 expression in dense breast tissue [46] and, subsequently, more effective inhibition of COX-2, as COX-2 might be key to driving mammary carcinogenesis [47,48]. Interestingly, a recent clinical trial with an 8-year follow-up suggested that COX-2 inhibitors were associated with poorer survival among breast cancer patients, especially those with a low expression level of COX-2 [49]. Therefore, it will be of interest to study the effect of non-aspirin NSAIDs among women with high COX-2 expression in the breast, regardless of a diagnosis of breast cancer. Our results did, however, not support that non-aspirin NSAID use may impact the risk of breast cancer with different molecular subtypes.

The strengths of this study include the large-scale population-based design for the analysis of the general Swedish female population and rich information about prescribed medications, cancer characteristics, and previous diagnoses, which enabled us to study the exclusive or non-exclusive use of prescribed aspirin and non-aspirin NSAIDs. An additional strength is the large sample size and rich data on breast density and other known risk factors of breast cancer in the Karma cohort.

A few limitations should be noted. First, the Prescribed Drug Register did not include medications purchased over the counter (OTC) or used in hospitals or nursing homes. Although ibuprofen is among the top-selling OTC drugs [50], it is sold only in small packages and at a higher price in Sweden [51]. Reassuringly, the proportion of such usage is likely small for aspirin. It is estimated that 95% of all aspirin used were covered in this register with another 4% used in hospitals or nursing homes and 1% OTC [52]. Regardless, we believe such misclassification of the exposure is nondifferential to the outcome, i.e., the percentage of misclassified use of NSAIDs does not relate to the later risk of developing breast cancer, and, therefore, would most likely have led to an underestimation of the associations of interest. That said, such misclassification may explain the null association observed between aspirin and breast cancer. However, the misclassification should be greater for non-aspirin NSAIDs, of which we observed a modest association with breast cancer. Second, our analysis was limited to the Swedish female population and may not be generalizable to other populations. Therefore, more studies in diverse demographic and geographical settings are needed to confirm our findings. Further, information on smoking, BMI, and the use of hormonal contraceptives was not complete before the year when these variables were included in the Swedish Medical Birth Register in the analysis of the general female population. This is, however, not likely to create differential misclassification between cases and controls. Lastly, given the limited number of cases in some of the subgroup analyses, we could not fully rule out the possibility of finding by chance. Studies with larger sample sizes are therefore needed to validate some of the subgroup results.

This study uses nationwide register-based and self-reported data from the entire female population and a mammographic screening cohort in Sweden and provides further evidence on the association between the use of NSAIDs and the risk of breast cancer by the stage and molecular subtype of cancer, breast density, and benign breast disorder.

## 5. Conclusions

In conclusion, we did not find strong evidence to support an association between the use of NSAIDs and the risk of breast cancer.

## Figures and Tables

**Table 1:** Characteristics of women with incident breast cancer (cases) and their individually matched breast cancer-free women (controls) in an analysis of the general female population in Sweden, 2006–2015.

Characteristics	Cases	Controls ^1^
Total number	56,480	282,400
Age at index date		
Mean (SD)	62 (13)	62 (13)
Range	21–102	21–103
Year of birth		
1900–1939	13,099 (23.2%)	65,495 (23.2%)
1940–1949	17,163 (30.4%)	85,815 (30.4%)
1950–1959	13,026 (23.1%)	65,130 (23.1%)
1960–1987	13,192 (23.4%)	65,960 (23.4%)
Region of birth		
Sweden	49,462 (87.6%)	244,263 (86.5%)
Other continents	1779 (3.1%)	10,230 (3.6%)
Other European countries	2532 (4.5%)	13,868 (4.9%)
Other Nordic countries	2707 (4.8%)	14,039 (5.0%)
Income ^2^		
Low	17,146 (30.4%)	91,071 (32.2%)
Medium	17,615 (31.2%)	86,000 (30.5%)
High	21,696 (38.4%)	105,201 (37.3%)
Unknown	23 (0.0%)	128 (0.0%)
Educational attainment		
Primary school (≤9 y)	13,597 (24.1%)	72,841 (25.8%)
Secondary school (10–12 y)	23,230 (41.1%)	119,354 (42.3%)
Postsecondary (>12 y)	19,284 (34.1%)	87,712 (31.1%)
Unknown	369 (0.7%)	2493 (0.9%)
Previous breast disorder		
No	50,704 (89.8%)	269,597 (95.5%)
Yes	5776 (10.2%)	12,803 (4.5%)
Previous malignancies ^3^		
No	51,046 (90.4%)	257,317 (91.1%)
Yes	5434 (9.6%)	25,083 (8.9%)
Cardiovascular disease		
No	51,165 (90.6%)	256,324 (90.8%)
Yes	5315 (9.4%)	26,076 (9.2%)
Musculoskeletal diseases		
No	49,903 (88.4%)	250,012 (88.5%)
Yes	6577 (11.6%)	32,388 (11.5%)
Systematic inflammatory diseases		
No	55,747 (98.7%)	278,047 (98.5%)
Yes	733 (1.3%)	4353 (1.5%)
Pain and fever		
No	53,192 (94.2%)	265,574 (94.0%)
Yes	3288 (5.8%)	16,826 (6.0%)
Charlson Comorbidity Index (CCI)		
CCI 0	44,996 (79.7%)	227,548 (80.6%)
CCI 1–3	10,574 (18.7%)	50,355 (17.8%)
CCI > 3	910 (1.6%)	4497 (1.6%)
Number of children		
0	8248 (14.6%)	37,832 (13.4%)
1–2	33,340 (59.0%)	161,524 (57.2%)
3 or more	14,892 (26.4%)	83,044 (29.4%)
Age at first childbirth		
Nulliparous/unknown	8258	37,829
13–19	4910 (10.2%)	26,665 (10.9%)
20–29	32,660 (67.7%)	170,421 (69.7%)
30–39	10,103 (21.0%)	45,015 (18.4%)
40–59	549 (1.1%)	2470 (1.0%)
Smoking ^4^		
N-Missing	37,065	186,332
1–9 cig/d	2811 (14.5%)	13,689 (14.2%)
10 cig or more/d	2232 (11.5%)	11,282 (11.7%)
Non-smoker	14,372 (74.0%)	71,097 (74.0%)
Use of hormonal contraceptive ^4^		
Yes	10,068 (17.8%)	48,346 (17.1%)
No/Unknown	46,412 (82.2%)	234,054 (82.9%)
Body mass index ^4^		
N-Missing	39,512	198,158
Mean (SD)	23 (3)	23 (4)
Range	11–53	10–59
Cumulative defined daily dose (DDD), mean (SD)		
Aspirin	865 (816)	880 (804)
Non-aspirin NSAIDs	145 (270)	150 (288)

SD, standard error. ^1^ Women who were born in the same year as the case woman and were still at risk for breast cancer at the time of the diagnosis of the case woman. Five controls per case were selected from each risk set. Numbers in controls do not reflect unique individuals because individuals could be included in more than one risk set. ^2^ Disposable income per consumption unit, categorized using according to tertiles of the whole population in each year. ^3^ Excluding nonmelanoma skin cancer. ^4^ Information was limited to women who gave birth in Sweden after 1982, i.e., 44% of the full population.

**Table 2:** Hazard ratios (95% confidence intervals) of exclusive and non-exclusive use of prescribed aspirin and non-aspirin NSAIDs in relation to the risk of breast cancer in an analysis of the general female population in Sweden, 2006–2015.

Exposure	Cases, N (%)	Controls, N (%)	Model 1 ^1^	*p*-Value (Model 1)	Model 2 ^2^	*p*-Value (Model 2)	Model 3 ^3^	*p*-Value (Model 3)
Exclusive use of NSAIDs								
No NSAIDs	32,984 (58.4%)	166,891 (59.1%)	1	-	1	-	1	-
Both	2146 (3.8%)	10,636 (3.8%)	1.04 (0.99, 1.09)	0.12	1.00 (0.95, 1.06)	0.86	1.14 (0.96, 1.34)	0.13
Only aspirin	2092 (3.7%)	10,718 (3.8%)	1.00 (0.95, 1.05)	0.95	0.97 (0.92, 1.03)	0.31	0.85 (0.69, 1.04)	0.12
Only non-aspirin NSAIDs	19,258 (34.1%)	94,155 (33.3%)	1.05 (1.03, 1.07)	<0.01	1.04 (1.02, 1.06)	<0.01	1.02 (0.98, 1.07)	0.31
Non-exclusive use of aspirin								
No	52,242 (92.5%)	261,046 (92.4%)	1	-	1	-	1	-
Yes	4238 (7.5%)	21,354 (7.6%)	1.00 (0.96, 1.04)	0.96	0.97 (0.94, 1.01)	0.15	1.00 (0.88, 1.14)	0.99
Per DDD increase in the average daily dose	-	-	0.98 (0.90, 1.06)	0.58	0.93 (0.85, 1.01)	0.08	0.90 (0.59, 1.38)	0.63
Previous use	1366 (2.4%)	6526 (2.3%)	1.05 (0.99, 1.11)	0.13	1.03 (0.96, 1.09)	0.42	1.08 (0.91, 1.28)	0.40
Recent use	2825 (5.0%)	14,597 (5.2%)	0.98 (0.94, 1.02)	0.27	0.95 (0.91, 0.99)	0.02	0.91 (0.75, 1.10)	0.33
Non-exclusive use of non-aspirin NSAIDs								
No	35,076 (62.1%)	177,609 (62.9%)	1	-	1	-	1	-
Yes	21,404 (37.9%)	104,791 (37.1%)	1.05 (1.03, 1.07)	<0.01	1.04 (1.02, 1.06)	<0.01	1.03 (0.99, 1.07)	0.18
Per DDD increase in the average daily dose	-	-	1.04 (0.92, 1.18)	0.56	0.98 (0.86, 1.12)	0.77	0.94 (0.69, 1.28)	0.68
Previous use	16,268 (28.8%)	79,645 (28.2%)	1.05 (1.02, 1.07)	<0.01	1.04 (1.01, 1.06)	<0.01	1.03 (0.98, 1.07)	0.22
Recent use	5083 (9.0%)	24,935 (8.8%)	1.05 (1.01, 1.08)	<0.01	1.03 (1.00, 1.07)	0.06	1.02 (0.95, 1.10)	0.54

DDD, defined daily dose. ^1^ Model 1 was adjusted for demographic factors, including the region of birth, educational attainment, and household income. ^2^ Model 2 was further adjusted for previous breast disorder, history of malignancies excluding nonmelanoma skin cancer, potential indications of NSAID use (i.e., cardiovascular disease, musculoskeletal diseases, systematic inflammatory diseases, and pain and fever), Charlson Comorbidity Index, number of children, and age at first childbirth, in addition to variables adjusted in model 1. ^3^ Model 3 was further adjusted for smoking, BMI, and the use of hormonal contraceptives, in addition to variables adjusted in model 2, among a subset of women with available data (44%).

**Table 3:** Hazard ratios (95% confidence intervals) of breast cancer in relation to the nonexclusive use of NSAIDs; analysis by cancer stage in an analysis of the general female population in Sweden, 2006–2015.

Stage	Exposure	Cases, N (%)	Controls, N (%)	Model 1 ^1^	*p*-Value (Model 1)	Model 2 ^2^	*p*-Value (Model 2)
Stage 0–2	No NSAIDs	21,385 (55.6%)	109,017 (56.7 %)	1	-	1	-
	Both	1606 (4.2%)	7834 (4.1%)	1.07 (1.01, 1.13)	0.03	1.02 (0.96, 1.09)	0.44
	Only aspirin	1435 (3.7%)	7373 (3.8%)	1.01 (0.95, 1.07)	0.82	0.98 (0.92, 1.05)	0.58
	Only non-aspirin NSAIDs	14,053 (36.5%)	68,171 (35.4%)	1.07 (1.04, 1.09)	<0.01	1.05 (1.02, 1.08)	<0.01
Stages 3–4	No NSAIDs	2019 (64.1%)	9210 (58.5%)	1	-	1	-
	Both	106 (3.4%)	735 (4.7%)	0.62 (0.50, 0.77)	<0.01	0.68 (0.54, 0.85)	<0.01
	Only aspirin	178 (5.7%)	876 (5.6%)	0.89 (0.75, 1.06)	0.20	0.91 (0.76, 1.10)	0.34
	Only non-aspirin NSAIDs	847 (26.9%)	4929 (31.3%)	0.76 (0.69, 0.83)	<0.01	0.80 (0.73, 0.88)	<0.01
Stage unclassified	No NSAIDs	9580 (64.5%)	48,664 (65.5%)	1	-	1	-
	Both	434 (2.9%)	2067 (2.8%)	1.10 (0.98, 1.22)	0.10	1.05 (0.94, 1.18)	0.39
	Only aspirin	479 (3.2%)	2469 (3.3%)	1.00 (0.90, 1.11)	0.97	0.96 (0.87, 1.07)	0.51
	Only non-aspirin NSAIDs	4358 (29.3%)	21,055 (28.4%)	1.07 (1.02, 1.12)	<0.01	1.06 (1.02, 1.11)	<0.01

^1^ Model 1 was adjusted for demographic factors, including the region of birth, educational attainment, and household income. ^2^ Model 2 was further adjusted for previous breast disorder, history of malignancies excluding nonmelanoma skin cancer, potential indications of NSAID use (i.e., cardiovascular disease, musculoskeletal diseases, systematic inflammatory diseases, and pain and fever), Charlson Comorbidity Index, number of children, and age at first childbirth, in addition to variables adjusted in model 1.

**Table 4:** Hazard ratios (95% confidence intervals) of breast cancer in relation to the exclusive use of NSAIDs; stratified analysis by mammographic density in an analysis of Karma participants, 2011–2019.

Breast Density ^1^	Exposure	Cases	Controls	Model 1 ^2^	*p*-Value (Model 1)	Model 2 ^3^	*p*-Value (Model 2)	Model 3 ^4^	*p*-Value (Model 3)
<9 cm^2^	None	67 (26.3%)	152,190 (32.6%)	1	-	1	-	1	-
	Both	18 (7.1%)	25,057 (5.4%)	1.36 (0.80, 2.30)	0.26	1.44 (0.83, 2.49)	0.20	1.39 (0.81, 2.36)	0.24
	Only aspirin	5 (2.0%)	9307 (2.0%)	1.05 (0.42, 2.61)	0.92	1.05 (0.42, 2.66)	0.91	1.02 (0.41, 2.56)	0.96
	Only non-aspirin NSAIDs	165 (64.7%)	280,120 (60.0%)	1.29 (0.97, 1.72)	0.08	1.35 (1.00, 1.81)	0.05	1.28 (0.96, 1.70)	0.10
9–40 cm^2^	None	219 (34.9%)	354,769 (37.4%)	1	-	1	-	1	-
	Both	33 (5.3%)	35,312 (3.7%)	1.24 (0.86, 1.81)	0.25	1.17 (0.79, 1.73)	0.42	1.19 (0.82, 1.73)	0.36
	Only aspirin	11 (1.8%)	14,072 (1.5%)	1.02 (0.55, 1.88)	0.95	0.97 (0.52, 1.8)	0.93	1.00 (0.54, 1.85)	0.99
	Only non-aspirin NSAIDs	364 (58.1%)	543,800 (57.4%)	1.04 (0.87, 1.23)	0.69	1.03 (0.86, 1.22)	0.78	1.02 (0.86, 1.21)	0.79
>40 cm^2^	None	177 (46.8%)	202,292 (40.8%)	1	-	1	-	1	-
	Both	11 (2.9%)	12,589 (2.5%)	0.87 (0.46, 1.61)	0.65	0.85 (0.45, 1.62)	0.63	0.79 (0.42, 1.48)	0.45
	Only aspirin	5 (1.3%)	6251 (1.3%)	0.78 (0.32, 1.92)	0.60	0.80 (0.32, 1.98)	0.63	0.78 (0.32, 1.92)	0.59
	Only non-aspirin NSAIDs	185 (48.9%)	274,395 (55.4%)	0.75 (0.61, 0.92)	<0.01	0.72 (0.58, 0.89)	<0.01	0.72 (0.59, 0.89)	<0.01

^1^ Mammographic density was measured by the STRATUS program as absolute dense area (cm^2^), presented as the mean value of left/right mammograms, mediolateral oblique (MLO) view. ^2^ Model 1 was adjusted for demographic factors, including the year of education, European ancestry, BMI, smoking, alcohol consumption, and physical activity. ^3^ Model 2 was further adjusted for potential indications of NSAID use (i.e., cardiovascular disease, musculoskeletal diseases, systematic inflammatory diseases, and pain and fever), in addition to variables adjusted in model 1. ^4^ Model 3 was further adjusted for hormonal and reproductive factors, including age at menarche, number of pregnancies, age at first childbirth, number of childbirths, ever use of contraceptives, ever use of hormonal replacement therapy, menopausal status at enrollment, family history of breast cancer, family history of ovarian cancer, and previous malignancies, in addition to variables adjusted in model 1.

## Data Availability

Restrictions apply to the availability of these data. Data were obtained from Statistics Sweden (scb.se), the National Board of Health and Welfare (Socialstyrelsen, socialstyrelsen.se), and Karma (karmastudy.org) and are fully available to researchers upon approved applications.

## Supplementary Materials

The following supporting information can be downloaded at: https://www.mdpi.com/article/10.3390/cancers15030692/s1, Table S1. Meta-analyses and randomized controlled trials regarding the use of NSAIDs and the risk of breast cancer; Table S2. Characteristics of women with incident breast cancer (cases) and matched breast cancer-free women (controls) in an analysis of the Karma cohort, 2011–2019; Table S3. Exclusive and non-exclusive use of aspirin and non-aspirin NSAIDs in relation to the risk of breast cancer, Karma, 2011–2019; Table S4. Clinical characteristics among women with incident breast cancer in the Swedish general population in an analysis of the general female population in Sweden, 2008–2015; Table S5. Exclusive use of NSAIDs in relation to the risk of breast cancer of specific molecular subtype in an analysis of the general female population in Sweden, 2006–2015; Table S6. Exclusive use of NSAIDs in relation to the risk of breast cancer stratified by previous breast disorders in an analysis of the general female population in Sweden, 2006–2015; Supplementary methods. ER, PR, and HER2 status in NKBC.
