# Supplementary material for: Use of Nonsteroidal Anti-Inflammatory Drugs and Risk of Breast Cancer: Evidence from a General Female Population and a Mammographic Screening Cohort in Sweden
Source: Cancers (Basel). 2023 Jan 23;15(3):692. doi: 10.3390/cancers15030692 (PMC9913077; doi:10.3390/cancers15030692)
Supplement: Supplementary file 1 [file cancers-15-00692-s001.zip › cancers-2185198-supplementary.pdf]

cancers

MDPI

Article

Use of Non-Steroidal Anti-Inflammatory Drugs and Risk of Breast Cancer: Evidence from a General Female Population and a Mammographic Screening Cohort in Sweden

Kejia Hu [1], Maria Feychting [1], Donghao Lu [1], Arvid Sjölander [2], Kamila Czene [2], Per Hall [2], Fang Fang [1,*]

[1] Institute of Environmental Medicine, Karolinska Institutet, Stockholm 17177, Sweden
[2] Department of Medical Epidemiology and Biostatistics, Karolinska Institutet, Stockholm 17177, Sweden
* Correspondence: fang.fang@ki.se; Tel.: +46-8-5248-6131

Supplementary materials

Table S1. Meta-analyses and randomized controlled trials regarding use of NSAIDs and risk of breast cancer 2

Table S2. Characteristics of women with incident breast cancer (cases) and the matched breast cancer-free women (controls), an analysis of Karma cohort, 2011-2019 2

Table S3. Exclusive and non-exclusive use of aspirin and non-aspirin NSAIDs in relation to risk of breast cancer, an analysis of the Karma participants, 2011-2019 5

Table S4. Clinical characteristics of women with incident breast cancer, an analysis of the general female population in Sweden, 2008-2015 6

Table S5. Use of NSAIDs in relation to risk of breast cancer of specific molecular subtype, an analysis of the general female population in Sweden, 2006-2015 7

Table S6. Exclusive use of NSAIDs in relation to risk of breast cancer, stratified analysis by previous breast disorder, an analysis of the general female population in Sweden, 2006-2015 8

Supplementary methods 9

Table S1. Meta-analyses and randomized controlled trials regarding use of NSAIDs and risk of breast cancer

Study	Included studies/participants	Years of publication /follow-up	Exposure	Effect size
**Meta-analyses**				
6. (Algra and Rothwell 2012)	15 case-control studies	1950-2011	Aspirin	0.88 (0.82, 0.95)
7. (Luo et al. 2012)	19 cohort studies,<br>13 case-control studies,<br>1 randomized clinical trial	1966-2011	Aspirin	0.86 (0.81, 0.92)
8. (Zhong et al. 2015)	17 cohort and 15 case-control studies	1977-2015	Aspirin	0.90 (0.85, 0.95)
9. (de Pedro et al. 2015)	23 case-control studies	Until 2013	Any NSAID	0.90 (0.88, 0.92)
10. (Cao and Tan 2020)	16 case-control studies	1939-2019	Aspirin	0.83 (0.78, 0.89)
11. (Ma et al. 2021)	27 cohort and 15 case-control studies	1989-2019	Aspirin	0.94 (0.91, 0.97)
9. (de Pedro et al. 2015)	24 cohort studies	Until 2013	Any NSAID	0.97 (0.94, 1.00)
10. (Cao and Tan 2020)	22 cohort studies	1939-2019	Aspirin	0.96 (0.91, 1.01)
**Randomized controlled trials**				
3. (Cook et al. 2005)	39,876 participants	Average 10 years	100 mg aspirin every other day	0.98 (0.87, 1.09)
4. (Cook et al. 2013)	33,682 participants	Median 18 years	100 mg aspirin every other day	0.98 (0.90, 1.07)

Table S2. Characteristics of women with incident breast cancer (cases) and the matched breast cancer-free women (controls), an analysis of Karma cohort, 2011-2019

Characteristics	Cases	Controls [1]
**Total number**	1260	1910154
**Year of birth** [2]		
1936-1949	467 (37.1%)	572498 (30.0%)
1950-1959	359 (28.5%)	510815 (26.7%)
1960-1969	350 (27.8%)	684145 (35.8%)
1970-1975	84 (6.7%)	142696 (7.5%)
**Age at index date**		
Mean (SD)	61 (10)	59 (9)
Range	40 - 81	40 - 81
**Age at index date**		
40-49	202 (16.0%)	378733 (19.8%)
50-59	362 (28.7%)	643517 (33.7%)
60-69	400 (31.7%)	553535 (29.0%)
70-81	296 (23.5%)	334369 (17.5%)
**Year of education at enrolment to Karma**		

Characteristics	Cases	Controls [1]
Gymnasium	363 (28.8%)	553686 (29.0%)
Other/unknown	120 (9.5%)	164150 (8.6%)
University	604 (47.9%)	958906 (50.2%)
Up to 9 years	173 (13.7%)	233412 (12.2%)
**European ancestry**		
Yes	1165 (92.5%)	1760890 (92.2%)
No	29 (2.3%)	45251 (2.4%)
Unknown	66 (5.2%)	104013 (5.4%)
**Body mass index at enrolment to Karma**		
16-24.9	664 (52.7%)	1034027 (54.1%)
25-29.9	388 (30.8%)	567093 (29.7%)
30-55	142 (11.3%)	208913 (10.9%)
Unknown	66 (5.2%)	100121 (5.2%)
**Smoking status at enrolment to Karma**		
Yes	663 (52.6%)	947304 (49.6%)
No	515 (40.9%)	838193 (43.9%)
Unknown	82 (6.5%)	124657 (6.5%)
**Alcohol consumption per week at enrolment to Karma**		
Non-drinker	193 (15.3%)	321600 (16.8%)
-10g/d	726 (57.6%)	1096565 (57.4%)
10g/d or more	255 (20.2%)	358483 (18.8%)
Unknown	86 (6.8%)	133506 (7.0%)
**Physical activity at enrolment to Karma**		
Low	401 (31.8%)	574493 (30.1%)
Medium	390 (31.0%)	590382 (30.9%)
High	368 (29.2%)	585614 (30.7%)
Unknown	101 (8.0%)	159665 (8.4%)
**Age at menarche**		
8-11	140 (11.1%)	221913 (11.6%)
12	234 (18.6%)	375398 (19.7%)
13	332 (26.3%)	491617 (25.7%)
14	271 (21.5%)	388513 (20.3%)
15-26	185 (14.7%)	287661 (15.1%)
Unknown	98 (7.8%)	145052 (7.6%)
**Number of pregnancies at enrolment to Karma**		
0	97 (7.7%)	155952 (8.2%)
1-3	847 (67.2%)	1222085 (64.0%)
>3	238 (18.9%)	414048 (21.7%)
Unknown	78 (6.2%)	118069 (6.2%)
**Age at first childbirth**		
13-24	334 (26.5%)	525186 (27.5%)
25-29	386 (30.6%)	561206 (29.4%)
30-34	216 (17.1%)	329745 (17.3%)
35-54	100 (7.9%)	150357 (7.9%)
Unknown	224 (17.8%)	343660 (18.0%)
**Number of childbirths at enrolment to Karma**		
0	145 (11.5%)	225093 (11.8%)
1-3	995 (79.0%)	1479192 (77.4%)
>3	41 (3.3%)	87834 (4.6%)

Characteristics	Cases	Controls [1]
Unknown	79 (6.3%)	118035 (6.2%)
**Ever use of oral contraceptives before enrolment to Karma**		
Yes	990 (78.6%)	1516997 (79.4%)
No	187 (14.8%)	256669 (13.4%)
Unknown	83 (6.6%)	136488 (7.1%)
**Ever use of hormonal treatment before enrolment to Karma**		
Yes	405 (32.1%)	491865 (25.8%)
No	770 (61.1%)	1293321 (67.7%)
Unknown	85 (6.7%)	124968 (6.5%)
**Menopause status at enrolment to Karma**		
Premenopausal	418 (33.2%)	775257 (40.6%)
Postmenopausal	842 (66.8%)	1134897 (59.4%)
**Breast cancer in first-degree relatives**		
Yes	249 (19.8%)	225469 (11.8%)
No	916 (72.7%)	1535337 (80.4%)
Unknown	95 (7.5%)	149348 (7.8%)
**Breast cancer in second-degree relatives**		
Yes	123 (9.8%)	150030 (7.9%)
No	626 (49.7%)	1040288 (54.5%)
Unknown	511 (40.6%)	719836 (37.7%)
**Ovarian cancer in the family**		
Yes	46 (3.7%)	70256 (3.7%)
No	1081 (85.8%)	1651801 (86.5%)
Unknown	133 (10.6%)	188097 (9.8%)
**Benign breast disorder**		
Yes	360 (28.6%)	401936 (21.0%)
No	813 (64.5%)	1377650 (72.1%)
Unknown	87 (6.9%)	130568 (6.8%)
**Other malignancies**		
Yes	121 (9.6%)	140240 (7.3%)
Unknown	1139 (90.4%)	1769914 (92.7%)
**Mammographic density categorization**		
<9 cm^2	255 (20.2%)	466674 (24.4%)
9-40 cm^2	627 (49.8%)	947953 (49.6%)
>40 cm^2	378 (30.0%)	495527 (25.9%)
**Cardiovascular disease**		
No	1130 (89.7%)	1735969 (90.9%)
Yes	130 (10.3%)	174185 (9.1%)
**Musculoskeletal diseases**		
No	1027 (81.5%)	1586218 (83.0%)
Yes	233 (18.5%)	323936 (17.0%)
**Systematic inflammatory diseases**		
No	1238 (98.3%)	1878286 (98.3%)
Yes	22 (1.7%)	31868 (1.7%)
**Pain and fever**		
No	970 (77.0%)	1483284 (77.7%)
Yes	290 (23.0%)	426870 (22.3%)

NSAIDs, non-steroidal anti-inflammatory drugs

[1] The case woman and women who were born in the same year as the case woman and still at risk for breast cancer at the diagnosis date of the case woman (controls) form a risk set. The number of controls does not reflect unique individuals because individuals could be included in multiple risk sets.
[2] The percentage of each category differs across cases and controls because the number of controls is not fixed between risk sets.

Table S3. Exclusive and non-exclusive use of aspirin and non-aspirin NSAIDs in relation to risk of breast cancer, an analysis of the Karma participants, 2011-2019

			Model1 [1]	Model2 [2]	Model3 [3]
**Exposure**	**Cases**	**Controls**	**HR (95% CI)**	**HR (95% CI)**	**HR (95% CI)**
**Exclusive use of NSAIDs**					
No NSAIDs	463 (36.7%)	709251 (37.1%)	1	1	1
Both	62 (4.9%)	72958 (3.8%)	1.11 (0.84, 1.45)	1.08 (0.82, 1.44)	1.07 (0.82, 1.41)
Only aspirin	21 (1.7%)	29630 (1.6%)	0.92 (0.59, 1.43)	0.91 (0.58, 1.42)	0.90 (0.58, 1.40)
Only non-aspirin NSAIDs	714 (56.7%)	1098315 (57.5%)	0.97 (0.86, 1.09)	0.97 (0.86, 1.09)	0.95 (0.85, 1.07)
**Non-exclusive use of aspirin**					
No	1177 (93.4%)	1807566 (94.6%)	1	1	1
Yes	83 (6.6%)	102588 (5.4%)	1.07 (0.85, 1.34)	1.06 (0.84, 1.34)	1.06 (0.84, 1.33)
Per DDD increase of average daily dose	-	-	1.09 (0.45, 2.66)	1.04 (0.42, 2.61)	1.06 (0.44, 2.59)
Previous use	52 (4.1%)	67921 (3.6%)	1.04 (0.78, 1.37)	1.02 (0.77, 1.36)	1.02 (0.77, 1.35)
Recent use	31 (2.5%)	34757 (1.8%)	1.14 (0.79, 1.63)	1.12 (0.77, 1.62)	1.13 (0.79, 1.62)
**Non-exclusive use of non-aspirin NSAIDs**					
No	484 (38.4%)	738881 (38.7%)	1	1	1
Yes	776 (61.6%)	1171273 (61.3%)	0.98 (0.88, 1.11)	0.98 (0.87, 1.1)	0.96 (0.86, 1.08)
Per DDD increase of average daily dose	-	-	1.12 (0.52, 2.43)	1.11 (0.49, 2.49)	1.02 (0.46, 2.24)
Previous use	669 (53.1%)	1014818 (53.1%)	0.98 (0.87, 1.10)	0.97 (0.86, 1.10)	0.96 (0.85, 1.08)

Exposure	Cases	Controls	Model1 [1] HR (95% CI)	Model2 [2] HR (95% CI)	Model3 [3] HR (95% CI)
Recent use	107 (8.5%)	156515 (8.2%)	1.02 (0.83, 1.26)	1.01 (0.82, 1.26)	1.00 (0.81, 1.23)

DDD, defined daily dose; HR, hazard ratio
[1] Model 1 was adjusted for demographic factors, including years of education, European ancestry, body mass index, smoking, alcohol consumption, and physical activity.
[2] Model 2 was further adjusted for potential indications for NSAIDs use such as cardiovascular disease, musculoskeletal diseases, systematic inflammatory diseases, and pain and fever, in addition to adjustment made in model 1.
[3] Model 3 was further adjusted for hormonal and reproductive factors, including age at menarche, number of pregnancies, age at first childbirth, number of childbirths, ever use of contraceptives, ever use of hormonal replacement therapy, menopausal status, family history of breast cancer, family history of ovarian cancer, and other malignancies, in addition to adjustment made in model 1.

Table S4. Clinical characteristics of women with incident breast cancer, an analysis of the general female population in Sweden, 2008-2015

	**Breast cancer cases**
**Total number**	46428
**Stage**	
Stage 0	5456 (11.8%)
Stage 1	15612 (33.6%)
Stage 2	12094 (26.0%)
Stage 3	1816 (3.9%)
Stage 4	773 (1.7%)
Stage unclassified	10677 (23.0%)
**Estrogen receptor (ER)**	
Positive	33637 (72.4%)
Negative	5591 (12.0%)
Unclassified	7200 (15.5%)
**Progesterone receptor (PR)**	
Positive	28184 (60.7%)
Negative	10797 (23.3%)
Unclassified	7447 (16.0%)
**Human epidermal growth factor receptor 2 (HER2)**	
Positive	5140 (11.1%)
Negative	32340 (69.7%)
Unclassified	8948 (19.3%)
**Molecular subtype** [1]	

	Breast cancer cases
Luminal A	24149 (52.0%)
Luminal B1	4093 (8.8%)
Luminal B2	3344 (7.2%)
Triple-negative	3399 (7.3%)
Her2-enriched	1620 (3.5%)
Unclassified	9823 (21.2%)

[1] Breast cancer subtypes were classified based on estrogen receptor (ER), progesterone receptor (PR), and human epidermal growth factor receptor 2 (HER2). Luminal A: ER+, PR+, HER2-; Luminal B1: ER+, PR-, HER2-; Luminal B2: ER+, HER2+; Triple-negative: ER-, PR-, HER2-; HER2-enriched: ER-, PR-, HER2+; the rest were unclassified, mostly because of undetermined HER2 status.

Table S5. Use of NSAIDs in relation to risk of breast cancer of specific molecular subtype, an analysis of the general female population in Sweden, 2006-2015

Subtype [1]	Exposure	Cases	Controls	Model1 [2] HR (95% CI)	Model2 [3] HR (95% CI)
Luminal A	No NSAIDs	12232 (50.7%)	62014 (51.4%)	1	1
	Both	1154 (4.8%)	5568 (4.6%)	1.07 (1.00, 1.14)	1.05 (0.98, 1.12)
	Only aspirin	923 (3.8%)	5021 (4.2%)	0.94 (0.87, 1.01)	0.93 (0.86, 1.01)
	Only non-aspirin NSAIDs	9840 (40.7%)	48142 (39.9%)	1.05 (1.02, 1.08)	1.04 (1.01, 1.07)
Luminal B1	No NSAIDs	2080 (50.8%)	10597 (51.8%)	1	1
	Both	209 (5.1%)	1047 (5.1%)	1.05 (0.89, 1.23)	1.07 (0.90, 1.25)
	Only aspirin	208 (5.1%)	1006 (4.9%)	1.07 (0.91, 1.26)	1.09 (0.92, 1.29)
	Only non-aspirin NSAIDs	1596 (39.0%)	7815 (38.2%)	1.05 (0.98, 1.13)	1.06 (0.98, 1.14)
Luminal B2	No NSAIDs	1772 (53.0%)	8894 (53.2%)	1	1
	Both	130 (3.9%)	626 (3.7%)	1.07 (0.88, 1.31)	1.07 (0.87, 1.32)
	Only aspirin	97 (2.9%)	504 (3.0%)	0.99 (0.79, 1.24)	1.01 (0.79, 1.27)
	Only non-aspirin NSAIDs	1345 (40.2%)	6696 (40.0%)	1.02 (0.94, 1.11)	1.02 (0.93, 1.10)
Triple-negative	No NSAIDs	1757 (51.7%)	9034 (53.2%)	1	1
	Both	143 (4.2%)	740 (4.4%)	1.00 (0.82, 1.21)	0.97 (0.80, 1.19)
	Only aspirin	115 (3.4%)	718 (4.2%)	0.82 (0.66, 1.01)	0.80 (0.64, 0.99)
	Only non-aspirin NSAIDs	1384 (40.7%)	6503 (38.3%)	1.11 (1.02, 1.20)	1.09 (1.00, 1.18)
Her2-enriched	No NSAIDs	830 (51.2%)	4248 (52.4%)	1	1
	Both	50 (3.1%)	316 (3.9%)	0.82 (0.60, 1.13)	0.76 (0.55, 1.06)
	Only aspirin	62 (3.8%)	262 (3.2%)	1.22 (0.91, 1.63)	1.15 (0.84, 1.56)

**Subtype** [1]	**Exposure**	**Cases**	**Controls**	**Model1** [2] **HR (95% CI)**	**Model2** [3] **HR (95% CI)**
	Only non-aspirin NSAIDs	678 (41.9%)	3274 (40.4%)	1.06 (0.95, 1.19)	1.05 (0.94, 1.18)
Unclassified	No NSAIDs	14313 (72.0%)	72104 (72.6%)	1	1
	Both	460 (2.3%)	2339 (2.4%)	1.01 (0.90, 1.12)	0.95 (0.85, 1.06)
	Only aspirin	687 (3.5%)	3207 (3.2%)	1.10 (1.00, 1.20)	1.03 (0.94, 1.13)
	Only non-aspirin NSAIDs	4415 (22.2%)	21725 (21.9%)	1.03 (0.99, 1.08)	1.03 (0.99, 1.07)

[1] Subtypes were classified based on estrogen receptor (ER), progesterone receptor (PR), and human epidermal growth factor receptor 2 (HER2). Luminal A: ER+, PR+, HER2-; Luminal B1: ER+, PR-, HER2-; Luminal B2: ER+, HER2+; Triple-negative: ER-, PR-, HER2-; HER2-enriched: ER-, PR-, HER2+; the rest were unclassified, mostly because of undetermined HER2 status.

[2] Model 1 was adjusted for demographic factors, including the region of birth, educational attainment, and household income.

[3] Model 2 was further adjusted for previous breast disorder, history of malignancies excluding non-melanoma skin cancer, potential indications for NSAIDs use (e.g., cardiovascular disease, musculoskeletal diseases, systematic inflammatory diseases, and pain and fever), Charlson Comorbidity Index, number of children, and age at first childbirth, in addition to the adjustment made in model 1.

Table S6. Exclusive use of NSAIDs in relation to risk of breast cancer, stratified analysis by previous breast disorder, an analysis of the general female population in Sweden, 2006-2015

**Previous breast disorder**	**Exposure**	**Cases, N (%)**	**Controls, N (%)**	**Model1** [1] **HR (95% CI)**	**Model2** [2] **HR (95% CI)**
**No**	No NSAIDs	29713 (58.6%)	160525 (59.5%)	1	1
	Both	1904 (3.8%)	10025 (3.7%)	1.02 (0.97, 1.08)	1.01 (0.96, 1.07)
	Only aspirin	1941 (3.8%)	10328 (3.8%)	1.01 (0.95, 1.06)	0.99 (0.94, 1.04)
	Only non-aspirin NSAIDs	17146 (33.8%)	88719 (32.9%)	1.05 (1.03, 1.07)	1.05 (1.03, 1.08)
**Yes**	No NSAIDs	3271 (56.6%)	6366 (49.7%)	1	1
	Both	242 (4.2%)	611 (4.8%)	0.97 (0.63, 1.49)	0.95 (0.60, 1.50)
	Only aspirin	151 (2.6%)	390 (3.0%)	0.76 (0.43, 1.32)	0.76 (0.43, 1.32)
	Only non-aspirin NSAIDs	2112 (36.6%)	5436 (42.5%)	0.85 (0.72, 1.01)	0.85 (0.72, 1.01)

[1] Model 1 was adjusted for demographic factors, including the region of birth, educational attainment, and household income.

[2] Model 2 was further adjusted for previous breast disorder, history of malignancies excluding non-melanoma skin cancer, potential indications for NSAIDs use (e.g., cardiovascular disease, musculoskeletal diseases, systematic inflammatory diseases, and pain and fever), Charlson Comorbidity Index, number of children, and age at first childbirth, in addition to the adjustment made in model 1.

Supplementary methods

ER, PR and HER2 status in NKBC

As the Cancer Register and NKBC are independent registers, the date of breast cancer diagnosis can be slightly different between registers. We checked among the 48,249 women with a diagnosis of breast cancer in both registers and found the difference between two diagnosis dates to be within 90 days for 47,288 (98%) women. We therefore linked the information from NKBC to the case woman if the diagnosis in NKBC was within 90 days before or after the date of diagnosis in the Cancer Register. If there was more than one diagnosis in NKBC, we kept the record closest to the date of diagnosis in the Cancer Register. We combined multiple records within the same date and defined the status of ER, PR, and HER2 as "positive" if any of the records indicated "positive", and "negative" if any of the records indicated "negative". If neither "positive" nor "negative" was reported, the status was defined as "unclassified". HER2 status was derived from immunohistochemistry (IHC) and gene amplification using fluorescence in situ hybridization (FISH) test. A "positive" status was determined if the IHC result was "3+" or if the gene copy result was "amplified". A "negative" status was determined if the immunohistochemistry result was "1+" or if the gene copy result was "not amplified". A status of "unclassified" was determined for the rest.
